# Functional Neuronal Topography: A Statistical Approach to Micro Mapping Neuronal Location

**DOI:** 10.3389/fncir.2018.00084

**Published:** 2018-10-16

**Authors:** Angela Jacques, Alison Wright, Nicholas Chaaya, Anne Overell, Hadley C. Bergstrom, Craig McDonald, Andrew R. Battle, Luke R. Johnson

**Affiliations:** ^1^Translational Research Institute, Brisbane, QLD, Australia; ^2^Institute for Biomedical Innovation, Queensland University of Technology, Brisbane, QLD, Australia; ^3^School of Psychology and Counseling, Queensland University of Technology, Brisbane, QLD, Australia; ^4^Faculty of Health Science and Medicine, Bond University, Gold Coast, QLD, Australia; ^5^Psychological Science Department, Vassar College, Poughkeepsie, NY, United States; ^6^Department of Psychology, George Mason University, Fairfax, VA, United States; ^7^School of Biomedical Sciences, Queensland University of Technology, Brisbane, QLD, Australia; ^8^Translational Research Institute, School of Medicine, The University of Queensland Diamantina Institute, Brisbane, QLD, Australia; ^9^Department of Psychiatry and Center for the Study of Traumatic Stress, Uniformed Services University School of Medicine, Bethesda, MD, United States

**Keywords:** microanatomy, memory, network, allocation, cluster, topography, heat maps, amygdala

## Abstract

In order to understand the relationship between neuronal organization and behavior, precise methods that identify and quantify functional cellular ensembles are required. This is especially true in the quest to understand the mechanisms of memory. Brain structures involved in memory formation and storage, as well as the molecular determinates of memory are well-known, however, the microanatomy of functional neuronal networks remain largely unidentified. We developed a novel approach to statistically map molecular markers in neuronal networks through quantitative topographic measurement. Brain nuclei and their subdivisions are well-defined – our approach allows for the identification of new functional micro-regions within established subdivisions. A set of analytic methods relevant for measurement of discrete neuronal data across a diverse range of brain subdivisions are presented. We provide a methodology for the measurement and quantitative comparison of functional micro-neural network activity based on immunohistochemical markers matched across individual brains using micro-binning and heat mapping within brain sub-nuclei. These techniques were applied to the measurement of different memory traces, allowing for greater understanding of the functional encoding within sub-nuclei and its behavior mediated change. These approaches can be used to understand other functional and behavioral questions, including sub-circuit organization, normal memory function and the complexities of pathology. Precise micro-mapping of functional neuronal topography provides essential data to decode network activity underlying behavior.

## Introduction

Following Cajal’s identification of the neuron as the fundamental functional unit of the nervous system (López-Muñoz et al., 2006), the field of neuroscience has endeavored to understand how neurons operates in local groups (ensembles) and distributed networks to bring about behavior. Cajal (1894) proposed a theory that memory storage requires the formation of new connections between neurons in the brain. How neurons and their 1000s of synaptic connections act together to encode a memory was first conceptualized by Hebb (1949) as neuronal ensembles that both spatially and temporally act together to encode a component of the memory. Since these foundational anatomical and theoretical works, newer studies involving fluorescent imaging and electron microscopy have since provided growing evidence for the modification of neuronal synapses as a result of information storage, now known as synaptic plasticity (Kandel, 2001; Korb and Finkbeiner, 2011). Thus, at the sub-cellular level knowledge of mechanisms of memory encoding is more established, in contrast at the neuronal ensemble level memory encoding mechanisms are not yet understood. Some functional evidence for Hebbian reverberatory networks connecting ensembles of neurons (Hebb, 1949) has been identified in memory circuits (Johnson et al., 2008, 2009; Josselyn et al., 2017). However, key challenges in neuroscience remain around how neurons collectively undergo plasticity in ensembles to encode memories and behaviors. Aspects of neural ensemble activity has been demonstrated in Hippocampus (Nakamura et al., 2010) and Caudate (Barbera et al., 2016) and in Amygdala (Johnson et al., 2008, 2009; Rogerson et al., 2014; Davis and Reijmers, 2017; Josselyn et al., 2017; Josselyn and Frankland, 2018). A key challenge in the neuroscience of memory is in identifying which neurons have been allocated to the memory trace and which have not, while some progress has been made (Bergstrom et al., 2008, 2011, 2013a,b; Bergstrom and Johnson, 2014; Mayford, 2014; Rogerson et al., 2014; Frankland and Josselyn, 2015; Bergstrom, 2016; Josselyn and Frankland, 2018), new techniques and approaches for understanding microanatomy are needed. This aim can be aided by the development of methods and approaches to help reliably identify and quantify systematic topographies of neurons allocated to specific memory traces.

Here, we developed a method for topographical analysis and measurement of neurons allocated to memory traces. We have applied this method to study aspects of the neurobiological encoding of fear memory. We termed this method “neuronal topographic density mapping” and have devised it to identify and map the degree of stability within a micro-topography of neurons encoding Pavlovian fear memory across different animals undergoing fear memory acquisition or extinction. The methods, described in detail below, were developed over multiple studies, investigating the location and distribution of neurons activated in fear memory in amygdala (LeDoux et al., 2006; Haranhalli et al., 2007; Bergstrom et al., 2011, 2013a,b; Johnson et al., 2012). For illustrative purposes and to expand on the scope of these techniques, we employed a small data set drawn from the study of **a**ctivity-**r**egulated **c**ytoskeleton-associated protein (Arc) expression in prefrontal cortex.

In our studies to date, we have investigated the micro-topography of memory using Pavlovian fear conditioning. In Pavlovian or classical fear conditioning a mild foot shock [unconditioned stimulus (US)] is temporally paired with an auditory tone or comparable visual stimuli [conditioned stimulus (CS)] (Johnson et al., 2012; Bergstrom et al., 2013a; Bergstrom and Johnson, 2014). The animal learns to associate the US with the CS and exhibits typical behaviors including freezing, typical of fear/threat behavior [described extensively by other authors (LeDoux, 2000; Fanselow and Gale, 2003; Johnson et al., 2012; Josselyn and Frankland, 2018)]. We measured neurons expressing plasticity associated proteins identified by immunocytochemistry. Other functional protein and RNA expression in neurons and glia can also be used with this approach. Differences were tested in the localization of neurons among the conditioned memory groups. We have provided a methodological approach to produce topographic neuron data from brain within precisely aligned anatomical regions. This approach enables investigation of the topographic patterns of neurons expressing plasticity associated proteins in the associative fear memory formation and its extinction. We propose that this method can also be used in the reproduction of neuronal density maps with regard to many forms of neuroscience data for example, drug treatments, stress and addiction or neurodegenerative disorders.

Our methodological approach to neuron topography, described here, provides useful advantages for localizing function across behavioral conditions. Other analysis methods to measure topography also provide useful topographic data. For example, Nakamura et al. (2010) identified that memory activated neurons formed small anatomical clusters in hippocampus during place preference formation, which was identified using a cluster analysis approach. Recent studies by Barbera et al. (2016) used measures of neuronal clustering of medium spiny neurons to predict locomotive states of behavior in mice. They reported that behavioral decoding accuracy improved using spatially distinct neural clusters over single neurons (Barbera et al., 2016).

Recent advances using *in vivo* optical methods including calcium imaging have provided a rich source of complex micro anatomical and dynamic neuronal data, including in awake behaving subjects (Ohki and Reid, 2014; Romano et al., 2017; Castanares et al., 2019). Recent analysis approaches for these data include the method developed by Romano and associates, to analyze neuronal population dynamics (Romano et al., 2017). Additional recent whole brain imaging and analysis techniques by Kim et al. (2015, 2017), who developed a spatial IEG-based mapping technique as a method to view whole-brain activity. Furthermore, whole brain mapping methods have also been developed by Vousden et al. (2015) and Renier et al. (2016). Each of these methods provide the advantage of visualizing patterns of neural activity across distributed brain networks. The creation of neuronal quantitative topographic density maps, as described here, can be used for a variety of studies to pinpoint functional microcircuits in the brain.

Using our approach to mapping and measuring topography we have characterized the microanatomy and topography of neurons involved in different phases of memory, consolidation, reconsolidation, and extinction (LeDoux et al., 2006; Haranhalli et al., 2007; Bergstrom et al., 2008, 2011, Bergstrom et al., 2013a,b; Bergstrom and Johnson, 2014). These data have the potential to pinpoint neuronal topography patterns underlying memory encoding in the mammalian brain in normal and pathological situations (Johnson et al., 2012) and thereby facilitate current treatments for pathological memory disorders (Johnson et al., 2012). The generation of neuronal topographic density maps can be used to define and measure memory allocation within the brain.

Throughout this methodological report we provide details of the rationale, procedures and equipment needed to produce and analyze topographic neuronal data. In addition, within each methodological section we provide ‘*examples*’ from our own data in order to illustrate how the methods can be applied and used. The methodological approaches we describe here have wide applications for understanding and measuring neuronal topography. Applications include measuring the topography of neurons encoding different types of memory, different sensory stimuli, and motor behaviors.

## Methods

### Data Collection: Behavioral, Tissue, and Neuron Analysis in Preparation for Topographic Investigation

#### Run Behavioral Models

In order to produce and analyze functional neuronal topography data linked to behavior, an appropriate behavioral model is needed. Behavioral model can include a variety of learning and memory models, addiction models, social interaction models, and other behaviors of interest. In our case we have investigated in detail Pavlovian fear conditioning.

Pavlovian fear conditioning leads to the formation of associative memories. Synaptic plasticity, dependent upon phosphorylation of extracellular signal-regulated kinase (pMAPK) has been identified as critical in the formation of these memories in the lateral amygdala (LA) and medial prefrontal cortex (mPFC) (LeDoux, 2000; Fanselow and Gale, 2003; Johnson et al., 2012; Josselyn and Frankland, 2018).

**FIGURE 1:**
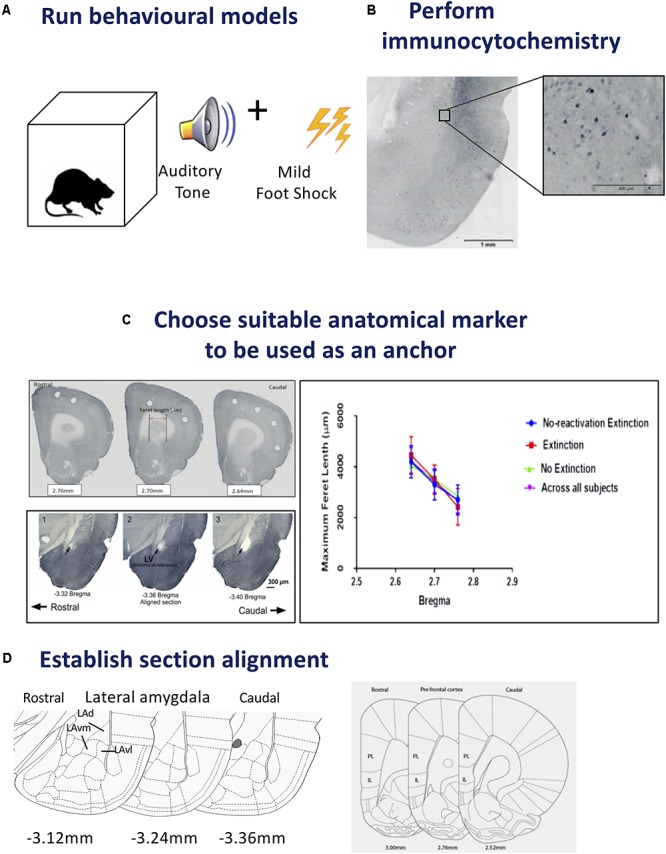
Steps for tissue sampling and measurement from behavioral data. **(A)** *Run behavioral models*. Any expression of a chosen behavior can be used as a model. In our example we have used Auditory Pavlovian fear conditioning. Behavioral testing was conducted with adult male Sprague-Dawley rats in acoustic classical fear conditioning chambers. A 0.6 mA foot shock with duration of 500 ms was paired with a tone of 5 kHz and 75 dB, 20 s in duration to produce an associative fear memory. **(B)** *Perform immunocytochemistry*. Avidin–biotin peroxidase complex method is demonstrated here. Sections from the lateral amygdala (LA) were labeled for Arc, scanned using a slide scanner and cropped at 2x magnification. Enlarged inset square shows Arc^+^ neurons in the dorsolateral portion of the LA at 20x magnification. Inverted gray scale images of fluorescent immunocytochemistry would also be suitable. **(C)** *Choose suitable anatomical marker to be used as an anchor*. The caudate putamen and lateral ventricle are two examples of anatomical landmarks, that we have used previously, and can be differentiated in serial sections for section alignment by Feret length within the ventile or between anatomical landmarks. Photomicrographs show three consecutive 60 μm sections across the rostrocaudal axis of the rat brain, depicting 2.76, 2.70, and 2.64 mm anterior to Bregma in the medial prefrontal cortex (mPFC). Feret diameter is shown – red arrow. Brain sections at Bregma coordinates –3.32, –3.36, and –3.40 mm posterior from Bregma were used to align the LA (Source: see Bergstrom et al., 2011). The maximum Feret length of the caudate putamen in the prefrontal cortex was shown to be statistically different across Bregma coordinates, animals and conditions. **(D)** *Establish section alignment*. The Rat Brain Atlas (Paxinos and Watson, 2007) is an important tool to assist alignment of sections. Schematic diagrams are shown depicting the regions of interest. The dorsolateral portion of the lateral amygdala (LAd), the ventromedial portion of the lateral amygdala (LAvm) and the ventrolateral portion of the amygdala (LAvl) are shown in three serial sections caudal from bregma –3.36 mm. The prelimbic (PL) and infralimbic (IL) cortex are represented by three serial sections caudal from bregma 2.52 mm. Brain Atlas diagrams are adapted from Paxinos and Watson (2007).

*Example:* The sample data set consisted of fear conditioned adult male Sprague-Dawley rats (RRID:RGD_5508397) (*n* = 40) that underwent behavioral procedures in standard Pavlovian fear conditioning chambers (Coulbourn Instruments, Allentown, PA, United States) (see **Figure 1A**). The US, a 0.6 mA foot shock with duration of 500 ms, was paired with the CS, a tone of 5 kHz and 75 dB (Digitech Professional Sound Level Meter^1^, 20 s in duration, to produce an associative memory. Three pairings were presented with an average 180 s inter-trial interval with total time in box of 10 min. Standard conditioning and behavioral testing procedures were followed (LeDoux et al., 2006; Haranhalli et al., 2007; Bergstrom et al., 2008, 2011, 2013a,b; Bergstrom and Johnson, 2014). The experimenter was blind to the experimental conditions when scoring freezing behavior, which was defined as a lack of movement except that required for respiration (LeDoux et al., 1988). Next, brains were prepared for histological analysis and measurement.

#### Perform Immunohistochemistry

Rats were transcardially perfused and brains were post-fixed in 4% PFA overnight then stored in 0.1 M phosphate buffered saline. Free-floating serial coronal sections (40 μm) of the mPFC and amygdala were prepared using a vibratome (M11000; Pelco easiSlicer, Ted Pella, Inc., Redding, CA, United States). Sections from the LA and prefrontal cortex were labeled for pMAPK and Arc activation using the avidin–biotin peroxidase method. Detailed immunocytochemical methods can be obtained from our previous reports (see Bergstrom et al., 2011, 2013a). Slides were scanned with an Olympus VS120 slide scanner and cropped at 2x magnification (see **Figure 1B**).

#### Choose Anatomical Anchor/Marker

Establishing anatomical alignment between regions of interest (ROI) is necessary for visual comparison of neuron density in neural images, for sectioning the ROI into micro regions for analysis, and for both quantitative and visual analysis of the data. Therefore, choosing an appropriate anatomical anchor is a key step. The anchor point should: (1) be a readily visible anatomical feature that is close in proximity to the ROI, (2) be stable across subjects and conditions, and (3) change shape rapidly and distinctly as the viewing plane changes, so that different planes of view can be discriminated clearly. These characteristics are identifiable microscopically and importantly can also be quantified (see **Figure 1C**).

*Example:* The amygdala and mPFC have been implicated in Pavlovian fear conditioning (Fanselow and Gale, 2003; Johnson et al., 2012; Lee et al., 2015). In a series of studies, we have focused on the amygdala and have used the opening of the Lateral Vertical (LV) as an anatomical anchor (LeDoux et al., 2006; Haranhalli et al., 2007; Bergstrom et al., 2008, 2011, Bergstrom et al., 2013a,b; Bergstrom and Johnson, 2014). The LV has proved a useful structure for the purpose because it meets the criteria outlined above: (1) the LV is close in proximity to the amygdala, (2) the LV changes rapidly in size along the longitudinal plane, (3) the LV is a stable anatomical feature, and (4) LV changes can be seen clearly, and measured, through the sequence of planes on which the brains were sectioned, enabling quantitative analysis of the changes section by section. In order to further demonstrate and measure the properties of the LV for landmark suitability, in addition to histological measurements, we made measurements of the LV with MRI. Here, the morphological properties of the LV, including its increase in diameter along the rostral-caudal axis, were confirmed *in vivo*, using three-dimensional T2-weighted MRI to quantify its area (Bergstrom et al., 2013a). This rapid change from rostral to caudal allows for precise quantitative section alignment from plane to plane. In our histological studies the morphology of the LV was reconstructed from five consecutive planes (Bregma -3.36 to -3.48). The coronal plane with the least variance between conditions was found at Bregma -3.36 in the rat (Paxinos and Watson, 2007), the entrance of the LV, so this was chosen as the most suitable anatomical anchor, in addition, it could be readily visualized and measured. At -3.36 mm Bregma, in addition to the LV it is also possible to identify the major anatomical structures of the ROI (the subnuclei of the LA). The choice of the LV as an anatomical anchor was therefore suitable because it is amygdala-centric, changes shape rapidly and clearly, and is stable across subjects (LeDoux et al., 2006; Haranhalli et al., 2007; Bergstrom et al., 2008, 2011, Bergstrom et al., 2013a,b; Bergstrom and Johnson, 2014).

We used the caudate putamen as an anatomical landmark to align sections in the prefrontal cortex (described below). Aspects of the caudate putamen met the criteria we previously set for landmark identification (see **Figure 1D**). Histological images were captured as virtual slide images (OlyVia; format.vsi) using a slide scanner (Olympus VS120). Capturing images with a slide scanner (used in this example) is an alternative approach to live capturing of neuron data with a microscope connected directly to Neurolucida as used in our previous published data (LeDoux et al., 2006; Haranhalli et al., 2007; Bergstrom et al., 2008, 2011, Bergstrom et al., 2013a,b; Bergstrom and Johnson, 2014). In this example, we used OlyVIA XV Image Viewer (Olympus Australia Pty Ltd., Vic, RRID:SCR_014342) to ascertain and measure images within a Bregma range that showed an alteration in the size of the caudate putamen. The caudate putamen becomes visible 2.7 mm anterior to Bregma, distinctly widens and lengthens in serial coronal sections across the rostrocaudal axis. Three consecutive sections (Bregma 2.7–2.58 mm) were aligned and verified across subjects and conditions by statistical comparison (ANOVA) of the Feret length (Walton, 1948) (the maximum Feret length or distance between two perpendicular tangents) was measured with Neurolucida 360 software (Neurolucida, MBF BioScience, Williston, VT, United States, RRID:SCR_001775) and analyzed with SPSS (IBM SPSS Statistics 23, WA, SCR_002865). A similar comparison of sections was calculated using z-scores from each maximum Feret measurement of the caudate putamen. No outliers were detected using ±3.0 standard deviation (SD). This principle includes 99.9% of values coming from the same normal distribution. Additionally, outliers can also be checked using online software tools, e.g., GraphPad Prism. Next, in order to test each Bregma point assignment was dissimilar and no difference existed between experimental conditions, paired *t*-tests were performed on the Feret measures. Each distance was found to be statistically different (example 2.76 mm Bregma; *p* = 0.000304). This data was used to help exclude misaligned sections due to natural or histological induced variations. This quantitative analysis approach can thus be used to assign sections to distinct groups maximizing alignment accuracy for subsequent neuronal topography measures.

#### Section Alignment

Quantitative topographical data was produced beginning with neuron identification and section alignment. While LV and caudate putamen changes can be observed through a sequence of many planes, the ROS may be rostral or caudal to this point. For this reason, the chosen landmark is used only as a point of reference. Sections are aligned manually using the landmark and working rostrally or caudally through the sequential Bregma coordinates using the measurement of width of each section as a guide. For example, Bregma 2.76 mm is 0.48 mm away from Bregma 3.24 mm; therefore, there will be 8 μm × 60 μm sections or 12 μm × 40 μm sections between the two Bregma coordinates. This highlights the need for precision when slicing and marking serial sections. Having mounted sections in the correct order on slides prior to labeling decreases time taken during this stage.

### Generate Topography in Preparation for Analysis

#### Create Contour

**FIGURE 2:**
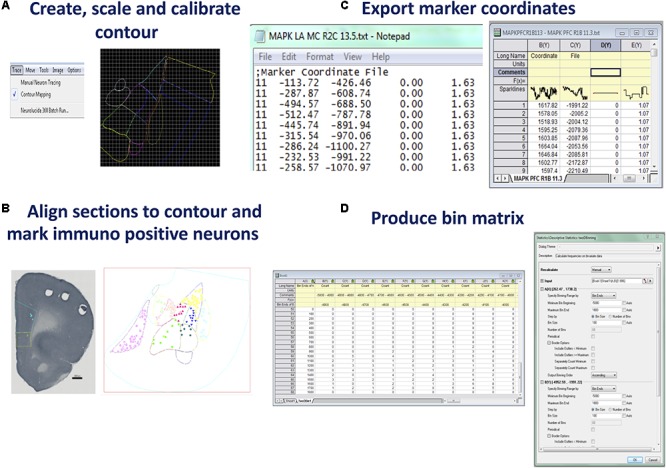
Steps for producing raw coordinate data from identified neurons. **(A)** C*reate, scale*, and *calibrate contour*. Neurolucida 360 (or an equivalent program) can be used to produce a nucleus or brain region contour from a rat brain atlas diagram. Using the contour mapping tool in Neurolucida 360 contours (in different colors) can be traced over a figure from an atlas. Lateral amygdala tracing shown was generated from Bregma –3.36 of Rat Atlas (Paxinos and Watson, 2007). **(B)** *Align section to contour and mark immuno positive neurons*. Prefrontal cortex section with contour overlaid. Immuno-positive neurons were marked within the contour. Saved data files can be opened in Neurolucida Explorer to gain data file information such as contour areas, Feret length measures, and neuron counts. Prelimbic contour and neurons were marked in aqua, infralimbic contour, and neurons marked in yellow. Once neurons are marked, Neurolucida Explorer (or equivalent) can import the data file to generate a contour and marker analysis, LA example shown. **(C)** *Export marker coordinates*. The x, y coordinates produced for each marked neuron are exported to an ASCII file which can be opened in graphing software such as Origin Pro (or equivalent). **(D)** *Produce bin matrix*. A data matrix is generated based on the area and density of marked neurons within the contour. Bin size is calculated using twice the area of the contour divided by the total number of neurons (De Smith et al., 2009). Once the x, y coordinates are highlighted in an Origin Pro (or equivalent) workbook, the 2D binning option under descriptive statistics is chosen. The bin ends and size can be manually entered into the dialog box once determined using the standard geospatial formula (De Smith et al., 2009).

In order to ensure consistency and precision in neuron counting across all subjects, a contour or tracing of the anatomical structure being investigated can be prepared in Neurolucida (NL) 360 (Neurolucida, MBF Bioscience, Williston, VT, United States). Prior to importing an image into NL for tracing, it is necessary to calibrate the image to approximate the dimensions of a single brain section bitmap image (cellSens software, Olympus, Notting Hill, VIC, Australia, RRID:SCR_014551). Within Neurolucida select > File, > Image open to allow the image to appear and select x and y calibration pixel size. These measurements are located in the image properties section in the cellSens program. Choose > Trace, > Contour Mapping in NL to begin the trace (see **Figure 2A**). The image lines may be enlarged using the zoom tool, to increase accuracy of the trace. Use the curser to trace around the selected area and > Close Contour when finished each area. This allows delineation of each section of the contour with a separate color using ‘User Line.’

#### Scale Contour

At this point it is essential to align the contour. The size of the tracing can be adjusted to fit the image using > Tools, > Adjust Scaling. Contour alignment must be consistent across all groups, prior to neuron counting. It is advisable to open several images to scale the contour, due to minor variation in dimensions across subjects.

#### Calibrate Contour

Very importantly, the contour is then calibrated to a constant point (0, 0 on the x, y axis) to preserve consistency of neuron marker coordinates. The reference point is displayed by selecting > Options, > Display Preferences, > View. In this window, the radius of the point can be set to a desired diameter. Apply the display grid setting and enlarge with the magnification tools as required. The contour is moved (using move tools) such that the 0,0 coordinates are placed in the superior left corner of the contour. Once in position the contour must not move or be resized for the duration of neuron counting across all groups to ensure the integrity of the quantitative data. Save contour as a data file.

#### Align Sections to Contour

Once the tracing has been saved > CTRL + S, a scanned and cropped image of a single neural section may be opened (> File, > Image Open, > calibrate pixel size) and the tracing can be overlaid using the move tools to move only the image. There may be some minor variation in the size and properties of each subject, driven by natural variation or variations introduced during tissue processing – therefore the contour must be aligned to each section. To align the section and the contour, select > Image, > Image Processing, and > Orientation (see **Figure 2B**). Options are provided for a mirror image, flip, 90 or 180° rotation of the image. Choose Arbitrary Rotation and use the arrows to alter the Rotation in Degrees.

#### Mark Immuno Positive Neurons

Once the section is aligned to the contour (or tracing), begin to mark neurons by choosing a marker from the marker toolbar located down the length of the left side of the screen. Right click the mouse button on the selected marker to rename, recolor or resize the marker. Elect to use a different color for markers in separate areas of the contour for ease of analysis at later stages of the process (see **Figure 2B**). Markers may be erased at any time during counting by > CTRL Z, or > Edit, > Undo, to remove the last placed marker. If mapping to determine the organization of synaptic markers within the neuropile, the same procedure should be followed for marking puncta (Radley et al., 2006).

*Note:* If mapping neurons using NeuroLucida directly connected to a microscope for live imaging, then, following contour tracing and neuron mapping, a final alignment of all data to be compared must be made before analysis of neuron spatial distribution. Contours with mapped neurons are rotated for matched alignment using the Neurolucida Contour Alignment function.

*Example:* A digital image of the ROI, the mPFC, was sourced from the rat brain atlas, 6^th^ Edn, 2007 (Paxinos and Watson, 2007, RRID:SCR_006369). Three locations, 3.3, 3.24, and 3.18 mm anterior to Bregma (Paxinos and Watson, 2007) were used for cell counting. This level was chosen as both the prelimbic and infralimbic cortices were represented at this point. Specific markers were recolored and renamed for each subregion to be mapped (**Figure 2B**).

#### Export Neurolucida ASCII File Into OriginPro (or Alternative)

Once all the neurons in the ROI are counted with the aligned contours, the marker coordinates (x, y, z), which Neurolucida has recorded relative to the nominated reference point, can be exported as an ASCII (plain text) file (see **Figure 2C**). To accomplish this, select > File, > Export Marker Coordinates and save the file. At this point it is also prudent to save the data file you have placed your makers on, by choosing > File, > Save Data File As. The Data file can be opened in Neurolucida Explorer > File, > open data file, > contour, > analysis, > markers and region analysis. This program provides a full synopsis of the contour areas, required for later mapping, perimeters, Feret measures, and neuron counts for each designated region. Once this information has been saved the neuron markers can be cleared in NL 360 using > Edit, > Select Objects. A window will open to the right of the screen where you can select Any Object, Only Markers, Select All, then press the Delete key. Choose > File, > Image Open to import a new section and begin the entire sequence again. Once two or more images are open, select > Image, > Image Organizer, to choose which images you will Show, Hide or Delete. Files can also be closed by selecting > File, > Close All Images. To analyze the data obtained the ASCII files can be opened in Microsoft Excel where the x and y coordinates are quickly accessed and can be cut and pasted into Origin Pro (see **Figure 1C**)^2^ . Alternatively, Origin Pro has the facility to open all files at once by choosing > File, > Import, > Multiple ASCII, and following the prompts to choose the files you wish to include in one density map. It is recommended to import only files from one behavioral condition at a time to reduce human error. Once coordinates are listed, select > Descriptive Statistics, > 2D Frequency Binning, which will require input of bin sizes (Alternatives to Origin Pro can also be used – see Discussion below).

#### Select Binned Data Parameters Within Origin Pro (or Alternative)

Data binning, also known as discretization, involves grouping data into bins in order to ascertain a quantitative understanding of neuronal distribution (Kerber, 1992). Developing an appropriate data matrix relies on the optimization of the dimensions of micro regions of data (bins). This part of the analysis should be well-considered and standardized in order to closely match the bin number and dimensions with the central experimental question being investigated and also to ensure the repeatability across subjects and experiments. The number of bins can be determined based on experimenter determined parameters or alternatively a formula can be applied to standardize the selection on bin numbers and to reduce any bias in bin number selection. An established formula for this type of spatial analysis is based on twice the expected frequency of items identified in a random field (2^∗^sampling area/*n*, where *n* = mean number of items to be counted, e.g., activated neurons) (De Smith et al., 2009). This method can be used to ensure an unbiased estimate of the optimal dimension of bins for sectioning the ROI into a matrix for data analysis. The neuron counts, and contour area measurements are obtained from the Neurolucida Explorer data. Once bin number has been calculated, the minimum bin beginning and maximum bin end for the x axis and y axis are adjusted to encompass the smallest and largest coordinates contained within the ASCII files. In Origin Pro, all Auto windows must be unchecked to allow manual input of data. The bin size is measured in micrometers squared (μm^2^). Once these measurements have been entered and the number of bins is calculated by the program, select > OK (see **Figure 2D**). This converts the data into an appropriate matrix, based on the area and density of the marked objects.

#### Produce Bin Matrix

The next step is to use the data from the calculated matrix of bins and their corresponding neuron counts for graphing and statistical analysis. The table of bins and neurons counts derived from Origin Pro (see **Figure 2D**) can now be copied into an Excel spreadsheet (or equivalent program). Repeat this process for each ASCII file obtained from one section, in one condition across all animals – this will be based on the section alignment for a specific “Bregma” coordinate – as described above. For validation purposes individual density maps can be produced at this point, for later comparison to the mean map. For an example see a range of 26 maps produced from raw values for each subject across four experimental conditions in comparison to mean maps in Figure 2 of Bergstrom et al. (2013a).

### Topographic Neuronal Density Maps (Heat Maps) and Analysis

#### Create Density Maps

**FIGURE 3:**
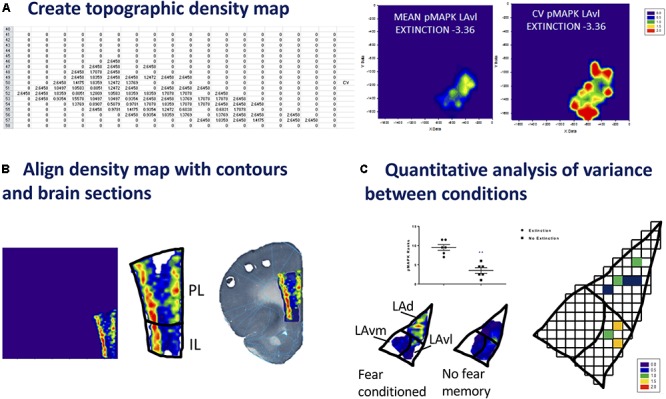
Steps for producing and analyzing topographical density maps. **(A)** *Create topographic density map*. A neuronal topographic density mean map is produced by transferring binned data from Excel to Sigma Plot (or equivalent software) (X data = x coordinates, Y data = y coordinates). Density maps can be created for each sub region. A coefficient of variance map can be prepared by dividing the standard deviation by the mean across all samples in one condition. Difference maps can also be created between conditions. The data matrix from Origin Pro (or equivalent) is transferred to a spreadsheet. This procedure is followed for each animal from a single condition/group. An average across all sheets produces the data for a mean density map. The standard deviation is calculated and divided by the mean, producing the data required for the coefficient of variance (CV) map. Example – topographic density (mean and CV) maps shown for Bregma –3.36, pMAPK^+^ neurons in the ventrolateral portion of the LA of rats that underwent extinction training (*n* = 7). **(B)** *Align density map with contour and brain sections*. To enhance visualization of specific neuronal subsets, density maps can be inserted into the contours or superimposed over brain sections. Density maps may be edited to change the styles, colors, font sizes, labels etc., providing alternatives conducive to individual requirements. Information regarding cell layers can be determined from visualizing the distribution of activated neurons as shown in the pMAPK labeling of the mPFC of rats that have undergone auditory fear conditioning (*n* = 7): mean map generated in Sigma Plot (or equivalent), map placed into contour, map overlaid on rat brain section. **(C)** *Quantitative analysis of variance between conditions.* A variety of statistical analysis can be performed to compare binned data such as Bonferroni correction, principal component analysis (PCA), false discovery rate (FDR), multiple discriminant analysis and mixed model ANOVA. Example of mean maps for the expression of pMAPK in the LA provides visual comparison between auditory fear conditioned (*n* = 6) and naïve (*n* = 7) rats. pMAPK^+^ ranks comparing extinction (*n* = 7) and no extinction (*n* = 5) groups within the ventrolateral portion of the LA *p* = 0.0022 (*t*-test, Mann–Whitney rank and SEM).

Using Excel, an average across all sheets can then be calculated – this is used to plot a graph of the mean for an experimental condition (see **Figure 3A**). In addition, from these combined and averaged data a coefficient of variance (*CV*) and other measures can be calculated. The mean and *CV* data can be used to create separate neuron topographic density ‘heat’ maps using graphing software SigmaPlot or OriginPro (SigmaPlot v 12.5, Systat Software, San Jose, CA, United States RRID:SCR_003210) (or alternatives). For producing a variety of graphs from the now binned data we have used SigmaPlot, however, other programs can be used. The data matrix, using individual subject data or averaged data from Excel, is transferred beginning in the third column of SigmaPlot. The x and y coordinates from Origin Pro are copied into columns one and two of Sigma Plot. In order to produce a colored neuron topographic density ‘heat’ map, select > Create Graph, > Contour Tool (see **Figure 3A**). The scale can be adjusted using the graph properties tool. The production of a neuronal topographic density ‘heat’ map is also possible using Origin Pro.

*Example:* We have used bin matrix data from neurons identified and marked in the prelimbic and infralimbic cortices and transferred this data to SigmaPlot. This data was used to produce both prelimbic (PL) and infralimbic (IL) mean neuron topographic density graph (heat maps). As described above, during the creation and alignment of the contour the 0, 0 coordinate was aligned to the superior left corner of the contour. The creation of an overlay was performed by aligning this same superior left landmark of the contour with the 0,0 coordinates as displayed on the SigmaPlot contour graph export. This process allowed aligned or registered heat maps from different animals to be combined into signed maps of mean data for initial qualitative analysis of the data sets. In our example we identified neurons activated during the recall of an extinguished fear memory – initial qualitative analysis of this data reveals increased neuron density within the deep layers of the PL and IL.

#### Align Maps With Contours and Sections

We recommend two methods to enhance visualization of specific neuronal subsets and gain visual information regarding distribution of activated neurons, for example, in relation to cell layer. The density maps can be inserted into the contours generated from an atlas, or alternatively superimposed over the original brain sections (see **Figure 3B**). To ensure ease of fit it is prudent to place a marker in the corner of each contour which can be removed prior to statistical analysis. Density maps may be edited in Sigma Plot to change the styles, colors, font sizes, labels etc., as requirements.

*Example:* Information regarding cell layers can be determined from visualizing the distribution of activated neurons as shown in the pMAPK labeling of the mPFC (see **Figure 3B**) of rats that have undergone auditory fear conditioning (*n* = 7): mean map generated in Sigma Plot (Systat Software). The map was placed into the prefrontal contour and overlaid onto a rat brain section.

#### Analysis of Binned Data

Graphing topographic neuron density data is an important step to provide visual evidence for changes in topography associated with behavioral and other experimental manipulations, as described above. However, when further evidence is needed to support conclusions of changes to neuronal topographic patterns then statistical analysis of the topographic data is required. Quantitative analysis can be performed with a variety of methods (discussed below) to compare topographical differences between conditions. Most common statistical software packages can be used for the analysis of topographical data. We have used GraphPad Prism 7 (GraphPad Software, Co., San Diego, CA, United States) for each of the below discussed methods, as well as linear regression and Pearson’s r coefficient which can also be collected for correlation between groups.

*Example:* To evaluate the bins in each data matrix, two-way ANOVA with a false discovery rate (FDR) correction for multiple comparisons was conducted. The discovered bins were termed micro-regions of interest (MORIs) and assigned a color to represent the density of neuronal cell bodies located in that position (see **Figure 3C**). *Post hoc* analysis of MROIs was conducted using corrected *t*-tests.

### Statistical Analysis of Topographic Neuron Density Data

In the next section, we describe statistical methods than can be applied to binned data sets of topographic data combined with behavioral manipulations to groups of experimental and control subjects. We also provide examples of application of statistical analysis from our own behavioral and neuronal topography data sets. The major challenge with the statistical analysis of multiple topographical binned data sets, combined with several experimental groups, is statistical error due to multiple comparisons. In order to best handle the analysis of topographical data we have investigated and utilized a variety of statistical approaches for large multiple comparison data sets – these include ANOVA and its variants; principal component analysis (PCA); and FDR correction (see **Table 1**). A very important step in performing statistical analysis of topographic data is to perform the statistical analysis in very close consultation with the Data produced from the topographic maps as described above. Through careful observation and consultation of the heat maps, derived from both individual animals and importantly behavioral group mean heat maps together with their measures of variance (*CV* maps), the most meaningful analyses can be performed and interpreted.

**Table 1:** Approaches for statistical analysis of neuron topographic data.

Method	Purpose	Advantage
ANOVA followed by Bonferroni corrected *t*-tests	To define where there is a significant difference in the data across conditions	Stringent control over type II errors
False discovery rate	To locate specific topographic regions of greatest variance across all conditions	Controls the expected rate of false rejection of the null hypothesis)
		Greater power
		Can be useful prior to correlational analysis
Principal component analysis	Identifies and ranks combinations of variables that account for variance within the data set	Extract meaningful patterns of neuronal variance related to the experimental manipulation
Multiple discriminant analysis	To visualize patterns within complex data sets	Determines how a set of continuous variables can discriminate groups
Mixed model ANOVA	Tests for differences between independent groups while	No adjustment for multiple comparisons is required
	using repeated measures to analyze topographic data	Accounts for random effects
	combined with experimental manipulations	^∗^GEE and ^∗∗^GAMM can be applied after, to accommodate non-linear relationships


*^∗^GEEs, generalized estimated equations; ^∗∗^GAMMs, generalized additive mixed models.*

#### ANOVA Followed by Bonferroni Corrected *t*-Tests

A question addressed in topographic data analysis is whether there is a significant difference in the data (e.g., number of activated neurons in the ROI) across all experimental conditions and in all ROI. One way to assess the overall difference in experimental manipulation is with analysis of variance (ANOVA), followed by a *post hoc t*-test with a correction for multiple comparisons (e.g., Bonferroni), among specific ROI and experimental groups to determine where the significance arises. Where multiple comparisons are necessary, a Bonferroni-type correction may be employed (see use in Bergstrom et al., 2011), however, it has the risk of being too strict and likely to sacrifice power in the attempt to exert stringent control over error. The potential for false negatives (type II errors) can be controlled effectively, while still retaining sufficient power, with FDR correction (Benjamini and Hochberg, 1995).

*Example:* We have analyzed topographic neuron density data from Pavlovian fear conditioning experiments in order to determine whether there were significant differences in topographic neuron density data across conditions by comparison of activated neuron density in each of the micro ROIs (46 bins) across all conditions via multiple comparisons (Bergstrom et al., 2013a). The mean numbers of activated neurons identified in the ROI from topographic data were used to conduct ANOVA across all conditions. Where a significant difference was found, planned contrasts between experimental and control groups were performed to assess where the differences lay (Bergstrom et al., 2013b). Multiple comparison tests involved three contrasts using one-way ANOVA. The first compared the fear conditioned and CS reactivated groups to the control groups: in this example, we compared box alone and CS (memory not reactivated groups). The second contrast was between the fear conditioned and CS reactivated groups and the third compared the box alone to the CS group. Having established a significant difference across conditions and located the main effect between experimental and control conditions, the next step was to locate the region of greatest variance in the ROI, requiring assessment of the differences in micro ROIs between groups (Bergstrom et al., 2013a). Furthermore, we also ran correlations with behavioral data as additional analysis (Bergstrom et al., 2013a).

#### False Discovery Rate (FDR)

Where the area under investigation has been sectioned into topographical units, each having its own data set, multiple ANOVAs on all topographical units may determine more precisely any variance between experimental conditions. FDR controls the expected rate of false rejection of the null hypothesis, by setting a parameter, the quotient q, as the “tolerable” FDR (Genovese et al., 2002). The *q*-value is used as an alternative to *p*-value when reporting significance, and while it may be set at a conventional level (0.05), a higher level may be reasonable (Genovese et al., 2002). FDR has been used effectively in neuroscientific studies (Genovese et al., 2002; Groppe et al., 2011; Bergstrom et al., 2013a; Bergstrom and Johnson, 2014). Once the region of greatest variance across all conditions is identified, follow up tests focus the investigation on the variance between experimental conditions, in those locations.

*Example:* We have previously successfully applied FDR for type II error minimization and identification of significance in specific topographic ROI in behavioral experiments (see Bergstrom et al., 2013a; Bergstrom and Johnson, 2014). In these studies, we conducted mass univariate ANOVAs to assess differences in neuron activation across all conditions in each of 46 bins. FDR correction was used, with the tolerable limit set at *q* = 0.1. Significant differences across conditions were found in certain micro ROIs (nine of 46 bins), so comparisons were performed on those particular data to locate (1) the effect of the experimental versus control groups and (2) the difference between two experimental groups (Bergstrom et al., 2013a; Bergstrom and Johnson, 2014). The *q*-values were mapped onto the topographical matrix (bins) to reveal the highly localized topography of neuronal activation. The spatial distribution of these points of significance was confirmed on visual analysis of the neuronal topographic density maps compiled from topographic data, and also reflected earlier findings (Bergstrom et al., 2011). Subsequent correlational analysis was used to confirm the relationship between the density of marked neurons and behavior.

#### Principal Component Analysis (PCA)

Another approach to topographical data with multiple ROI and group comparisons is PCA. PCA seeks to identify and rank combinations of variables that account for variance within the data set. PCA enables the relationships between these patterns of variables to be identified, tested and confirmed (Jolliffe, 2002). PCA has been applied by ourselves and others to address a variety of anatomical questions, for example in morphological studies of microglial cells (Soltys et al., 2005); and vagus nerves (Horn and Friedman, 2003); localization of sensory cells in the thalamus in facial recognition (Chapin and Nicolelis, 1999); the segregation of pyramidal neurons into morphological defined cell populations (Bergstrom et al., 2008); eye-tracking data (Bergstrom et al., 2016); and extensivley in MRI data (Lin F. et al., 2006).

*Example:* We have successfully applied PCA for the analysis of topographic neuronal density data activated in studies of Pavlovian fear conditioning. Activated neurons were mapped and the area sectioned into micro ROIs (bins) as described above, to produce a matrix of memory data (Bergstrom et al., 2011, 2013a). Ten components (of spatial data) were revealed, with one of these (SC1) being associated with the pattern of greatest difference (principal component score) in the spatial distribution of activated neurons between experimental conditions. SC1 displayed a unique pattern of activated neurons in a particular subnucleus of the amygdala (the LAd) across all brain samples in the experimental group. This was confirmed by *t*-test comparisons (Bonferroni corrected) of the bins with the most prominent loading values, and these also correlated with the area of highest density in the topographic analysis outlined above. That is, as described above, the statistical pattern could be confirmed by visual patterns seen in the neuronal topographic density maps generated by color-coding neuron densities. PCA has proved a useful statistical tool to extract meaningful patterns of variance related to the experimental manipulation, which could be confirmed by both comparison with visual representations of the data and Bonferroni corrected *t*-tests (Bergstrom et al., 2011, 2013a).

#### Multiple Discriminant Analysis (MDA)

Multiple discriminant analysis (MDA) is a method of visualizing patterns within complex data sets (Lin L. et al., 2006). With complex data, such a topographic data with many anatomical sub-regions and bins combined with multiple experimental conditions, where both location and distribution across area, are under investigation it can be important to identify patterns within this data set, in order to help understand and interpret the data. MDA can be used to determine how a set of continuous variables can discriminate groups (Bergstrom et al., 2013b), for example, how the pattern of neuron density in certain subnuclei (the independent or predictor variable) can predict the experimental condition the subject brain best fits into (the grouping or independent variable). MDA gives loading values (canonical variate correlation coefficients) that represent the relative contribution of each variable in a set of variables (a dimension) that discriminates groups from each other (see Lin L. et al., 2006; Bergstrom et al., 2013b).

*Example:* In one topography of Pavlovian fear memory study, we were interested in the relative contribution of lateral and basal amygdala (LA) subnuclei to the overall density of activated (pERK/MAPK expressing) neurons among each experimental condition (Bergstrom et al., 2013b). First, MANOVA was performed to examine the relationship among the subnuclei. Where a significant relationship was found, one-way ANOVA on each subnucleus tested for significant differences between conditions. Next, MDA was used to test the relative contribution of each subnucleus to the overall difference in density of activated neurons between conditions. The MDA revealed a single underlying pattern in density of activated neurons across lateral and basal amygdala subnuclei that discriminated the experimental and control groups. It also showed the subnucleus (the LAd) that contributed most to the overall difference between conditions. Having used MDA to help identify the region with the most significant contribution to the overall pattern of variance between conditions, it was possible to go further and explore more fine-grained details within the data. To confirm the pattern identified with MDA, *post hoc* comparisons with Bonferroni correction were performed, verifying the findings on the location and experimental condition of the greatest activation, and reinforcing ours and others previous findings about the predominance of LAd neural plasticity in fear memory (Rodrigues et al., 2004; Bergstrom et al., 2011).

#### Mixed Model ANOVA

The Mixed Model ANOVA also known as a Mixed Design ANOVA or a Split-Plot ANOVA, allows for testing for differences between independent groups (in functional topography experiment these will be the impendent behavioral groups, i.e., experiment and control groups) while using repeated measures (bins in topography experiments). Thus, the Mixed Model ANOVA can be employed for microanatomy data comprising neuron counts within bins contrasted across several independent groups. For our studies of functional neuronal topography, we typically derive 20–80 bins per animal comprising the within-group dependent variable. For the independent variable, several independent groups of animals are used including experiment and control groups. Mixed Models allow for the analysis of data from all locations and all animals in one analysis. Thus, Mixed Models a have strong potential for analysis of topographic data combined with experimental manipulations – such as behavioral or pharmacological manipulations. Using a Mixed Model analysis data between anatomic locations can be compared and no adjustment for multiple comparisons is required. Mixed Models can be thought as an advancement of ANOVA and regression models. One, very important but often overlooked, assumption of ANOVA/Regression, is that the data are independent of each other. Thus, the analysis cannot have the same individual represented twice in the same dataset. For example, measurements on LA have to be analyzed separately from infralimbic cortex.

Mixed models ANOVA offers a toolbox to account for the dependence of measurements taken on the same individual, by accounting for, so called, *random effects.* Random effects are variables for which we are not interested in the actual levels that we have sampled but on what they represent as a sample from a population. The most usual random effect would be the individual animal (for further definitions of *random effects* readers are directed to Fitzmaurice et al. (2004) and Zuur et al. (2007, 2009). Methods related to Mixed Model ANOVA that could also be applied to topographic data sets with is the generalized estimated equations (GEEs) and the generalized additive mixed models (GAMMs) which can accommodate non-linear relationships (for further information see, Zuur and Ieno, 2016 for GAMM and Fitzmaurice et al., 2004 on Mixed Model ANOVA and GEEs and their differences).

## Discussion

Understanding neural network organization and predicting memory and behavior from neural network functionality is a critical goal in the field of neuroscience. Although various imaging techniques are capable of large-scale analysis of functional brain regions, they are not suitable for imaging the spatial distribution, connectivity and stability of neurons at the micro-network level. The ability to accurately map, measure and compare neural network spatial properties, as described here, contributes to our fundamental awareness of the organization and structure of functional neural circuits. Classic cellular and molecular analysis of neuronal tissue assists in the identification of molecular machinery underlying behavior but does not answer questions relative to the fundamental organizational properties and their functional changes associated with behavior. We have developed a combined topographic and statistical approach for producing and analyzing micro-topographic data. This method provides clear visualization of the spatial organization and degree of consistent neuronal patterns across brains from individual subjects and in different experimental conditions.

Neuronal material used for topographic mapping can include both exogenously labeled, such as immunocytochemistry and *in situ* hybridization, as well as endogenous genetic labeling with green fluorescent protein (GFP) and other fluorescent probes. Consistency in labeling is important with regard to whichever neuron marking system is selected for topographic mapping. The statistical methods recommended and applied here allow for natural variation in measured populations. Nonetheless, reduction of variability will improve outcome consistency and statistical verifications. Marking neurons requires consistent labeling and consistent identification of neurons. To verify consistency, ideally experimenters blind to the experimental conditions are employed throughout or for verification checks of large data sets. The general principles outlined here for micro-topographic mapping can be applied to sectioned brain material as well as whole brain analysis approaches using CLARITY, CUBIC, or iDISCO. Three-dimensional analysis also requires focus and comparative measurements on specific anatomic ROS. Both 2D and 3D analysis ultimately requires localization and correlation of cellular activity with behavioral function using the approaches described here.

### Topographic Mapping

The first step in the approach to visual and quantitative analysis of functional neuronal topography between animals is to establish section alignment. Careful choice of an appropriate and stable landmark or anchor point associated with the ROS is essential (LeDoux et al., 2006; Haranhalli et al., 2007; Bergstrom et al., 2011, 2013a,b; Johnson et al., 2012; Bergstrom and Johnson, 2014). Identification of an anchor point which has rapid and distinct conformational change through sectional view planes will ensure success at this level. The second stage involves fitting a contour to the ROS, which ensures precision of the region in which the neurons will be counted, as well as consistency in the area across subjects. A limitation at this stage is small variation between sections from each subject, which can come from animal variations and also from histological processing, therefore care is needed to minimize variation. The contour must be fitted to each section with a degree of individual judgment. Specific brain regions, such as the hippocampus, may also significantly change in shape along the longitudinal axis and therefore a single contour is not feasible. An alternate approach entails producing a unique mean contour section for a specific data set. The rat brain atlas, developed by Paxinos and Watson in the 1980s (Paxinos and Watson, 2007), is one of the most established and detailed sources of anatomical coordinates available at this time. Other brain atlases are available and can also be used. In the Paxinos and Watson atlases, the depicted brain sections can appear up to 480 micrometers apart necessitating several brain sections to be mapped to individual atlas plates. Our method is therefore limited in part by the standardized atlas information currently available (Paxinos and Watson, 2007).

Prior to creating a contour an atlas image generally requires resizing, which can represent an amount of time spent making adjustments with various software packages. Due to the number of software packages used to produce the images, it is essential to note both the accepted file types (as listed in methods above) for compatibility as images are moved between programs. Furthermore, it is very important to note the numerical functions involved in any resizing, so that consistency is maintained. Computer processing speed and memory requirements must also be considered when using the large data files produced by slide scanning.

Free, open source programs are available for some procedures, making our described method economically viable to all. For example, Image J and FIJI (National Institutes of Health) can be substituted for some elements of the topographic mapping, as it is able to perform cell counts and export x,y coordinate data. Image J has many plugins available and runs in Java which is editable. Prior to this the contours must be calibrated to a zero point to facilitate precise individual comparisons. Once the coordinates have been exported a data matrix may be developed. Data bins are created using a geospatial analysis formula to establish unbiased bin dimensions. Open source programs are also available for this step requiring some degree of coding for specific features. QtiPlot (Free Software Foundation) is a free replacement for Origin and SigmaPlot. It will enable binning of x, y coordinates into a two-dimensional matrix and has contour generating capabilities for producing neuronal topographic density maps. Free online software for FDR analysis, as described above, is also available^3^. While we have outlined and described our methodical approach using a series of standalone commercial software packages for each of the steps descried, free software is also available making the methodical approaches described here freely available for all worldwide.

### Analysis of Topographic Data

Although we have presented several arguments for the use of binned data for micro-topographic analysis, there remains the opinion that discretization has limitations (MacCallum et al., 2002; Langseth, 2008). We have used both PCA as well as Mass Univariate ANOVA with FDR correction as a useful way to locate areas of most variance in complex data, and to confirm the qualitative data from our mean heat maps. This method assists in decreasing the reduction in power generated with Bonferroni procedures (Verhoeven et al., 2005). While we provide general guidance for analysis of binned micro-anatomical data sets, we advise the reader to liaise with statisticians to evaluate the methodical approaches described here with the chosen data analysis techniques for the analysis of unique data sets and research questions.

## Conclusion

Neuronal micro-topographic density maps can assist in defining specific brain regions involved in behavior. Statistically verified microanatomical mapping has the ability to advance our knowledge of the multi layered, complex organization of the brain and its cognitive systems. Our approach for the measurement and contrasting of neuronal topographic data in behavioral experiments has been successfully applied to the study of the microanatomy of memory formation. It has enabled us to visualize the spatial allocation of neurons activated during the acquisition of fear memories (LeDoux et al., 2006; Haranhalli et al., 2007; Bergstrom et al., 2011, 2013a,b; Johnson et al., 2012; Bergstrom and Johnson, 2014). We propose this method will prove advantageous to other forms of neuroscience, including the cellular basis of addiction; pathological memory models; pharmacological manipulations, and other forms of functional microanatomy (Johnson et al., 2012; Holmes and Singewald, 2013). Existing nuclei cataloged in brain atlases have been defined histologically, our approach allows for the identification of new functional micro-regions within established brain nuclei. By providing this walk-through tutorial we encourage further development of these goals.

## Ethics Statement

This study was approved by The University of Queensland Animal Ethics Committee.

## Author Contributions

AJ wrote paper, made figures, contributed to methods development. AW contributed to methods development. NC contributed to methods development. HB contributed to methods development and development of statistical approaches. CM contributed to methods development and development of statistical approaches. AO contributed to methods development. AB co-wrote the paper. LJ conceived and developed method, co-wrote the paper.

## Conflict of Interest Statement

The authors declare that the research was conducted in the absence of any commercial or financial relationships that could be construed as a potential conflict of interest.

## Footnotes

1 https://www.jaycar.com.au/pro-sound-level-meter-with-calibrator/p/QM1592

2 http://www.scientificcomputing.com/product-release/2014/10/origin-and-originpro-2015-data-analysis-and-graphing-software

3 sdmproject.com
